# The Gastric/Pancreatic Amylase Ratio Predicts Postoperative Pancreatic Fistula With High Sensitivity and Specificity

**DOI:** 10.1097/MD.0000000000000339

**Published:** 2015-01-26

**Authors:** Shuo Jin, Xiao-Ju Shi, Xiao-Dong Sun, Ping Zhang, Guo-Yue Lv, Xiao-Hong Du, Si-Yuan Wang, Guang-Yi Wang

**Affiliations:** From the Department of Hepatobiliary and Pancreatic Surgery (SJ, X-JS, X-DS, PZ, G-YL, X-HD, G-YW), Bethune First Hospital of Jilin University, Changchun 130021, Jilin; and Department of Surgery Intensive Care Unit (S-YW), Beijing Chao-yang Hospital Affiliated to Capital Medical University, Beijing, China.

## Abstract

This article aims to identify risk factors for postoperative pancreatic fistula (POPF) and evaluate the gastric/pancreatic amylase ratio (GPAR) on postoperative day (POD) 3 as a POPF predictor in patients who undergo pancreaticoduodenectomy (PD).

POPF significantly contributes to mortality and morbidity in patients who undergo PD. Previously identified predictors for POPF often have low predictive accuracy. Therefore, accurate POPF predictors are needed.

In this prospective cohort study, we measured the clinical and biochemical factors of 61 patients who underwent PD and diagnosed POPF according to the definition of the International Study Group of Pancreatic Fistula. We analyzed the association between POPF and various factors, identified POPF risk factors, and evaluated the predictive power of the GPAR on POD3 and the levels of serum and ascites amylase.

Of the 61 patients, 21 developed POPF. The color of the pancreatic drain fluid, POD1 serum, POD1 median output of pancreatic drain fluid volume, and GPAR were significantly associated with POPF. The color of the pancreatic drain fluid and high GPAR were independent risk factors. Although serum and ascites amylase did not predict POPF accurately, the cutoff value was 1.24, and GPAR predicted POPF with high sensitivity and specificity.

This is the first report demonstrating that high GPAR on POD3 is a risk factor for POPF and showing that GPAR is a more accurate predictor of POPF than the previously reported amylase markers.

## INTRODUCTION

Pancreaticoduodenectomy (PD) is a complex surgical procedure used to treat various benign and malignant diseases in the pancreatic head and periampullary region. Although recent advances in surgical techniques have reduced the rate of PD-associated mortality to <5%, the rates of postoperative morbidity remain high, ranging from 30% to 65%.^1–3^ Postoperative pancreatic fistula (POPF) is the most significant contributor to the high morbidity rate in patients who undergo PD. As the pancreatic fluid leaks into the surrounding tissues, complications such as abscess formation, delayed gastric emptying (DGE), and hemorrhage also occur.^3^ Consequently, POPF development is often associated with longer hospital stays and higher cost of health care.^1,4^

To improve clinical management after PD, there is a need for physicians to identify patients at high risk of POPF soon after the surgery. Previous studies have pinpointed several biochemical factors, including serum amylase level,^5^ drain fluid amylase level,^6^ combined serum albumin and leukocyte count,^7^ combined blood urea nitrogen (BUN) and serum albumin levels,^8^ and the persisting ratio of drain fluid amylase output,^9^ as POPF predictors. However, these markers often have low predictive accuracy, as demonstrated by their low positive predictive values (PPVs). We reasoned that these biomarkers, measured in the early phase after PD, may not be ideal POPF predictors because intrinsic and extrinsic factors, such as individual variations in pancreatic exocrine function, tissue response after surgery, and postoperative medication, may greatly influence their levels.

We believe that when pancreatic fluid flows back into the reconstructed digestive tract instead of draining through the external pancreatic drainage tube, it may cause tissue damage in the gastrointestinal anastomosis, and subsequently, POPF. Meanwhile, such back flow of pancreatic fluid may increase the amylase level in the gastric drain fluid and decreases in the amylase level in the pancreatic drain fluid. Consequently, patients with POPF may have a higher gastric/pancreatic amylase ratio (GPAR) than patients who do not have POPF. Pancreatic exocrine function can greatly vary in patients after PD, and this variation can lead to significant differences in the absolute levels of amylase in gastric or pancreatic drain fluid. Unlike absolute amylase levels, GPAR is less likely to be influenced by pancreatic exocrine function and can be a reliable indicator for the direction of pancreatic fluid flow. We further surmised that because the influence of postoperative tissue response or medication on amylase levels decreases over time,^10,11^ measurements on postoperative day (POD) 3 are more reliable than that on POD1. Therefore, we hypothesized that GPAR on POD3 may serve as an accurate predictor of POPF. In this study, we analyzed the association between POPF and multiple biomarkers – including previously known POPF risk factors and GPAR – evaluated GPAR as a predictor of POPF, and compared its predictive power to that of serum or drain fluid amylase levels, 2 conventional POPF predictors.

## MATERIALS AND METHODS

### Study Design and Patient Recruitment

This was a single-center study at the Division of Hepatobiliary and Pancreas Surgery, First Bethune Hospital of Jilin University, Jilin, China. All the patients were recruited prospectively. The inclusion criterion was having undergone PD (Child procedure with external pancreatic drainage) between August 2012 and September 2014. Patients with tumors invading the surrounding tissues, and therefore unsuitable for surgical treatment, were excluded from the study. We collected preoperative, intraoperative, and postoperative information from each patient. Preoperative information included age, sex, body mass index (BMI), history of diabetes, presentation of jaundice or low plasma protein, and preoperative jaundice treatment. Intraoperative information included pancreatic consistency, pancreatic duct diameter, and pancreatic anastomotic techniques used. Postoperative information included postoperative pathological examination results and volume of pancreatic and gastric drain fluid, pancreatic and gastric drain fluid amylase levels, and postoperative complications: hemorrhage, POPF, biliary fistula, DGE, and postoperative acute pancreatitis (Table 1). POPF was classified into grades A, B, and C according to the definition of the International Study Group of Pancreatic Fistula (ISGPF).^1,12^ The study protocol was designed according to Declaration of Helsinki guidelines and approved by the Institutional Ethics Committee of the First Hospital of Jilin University. Written informed consents were obtained from all patients.

**TABLE 1:**
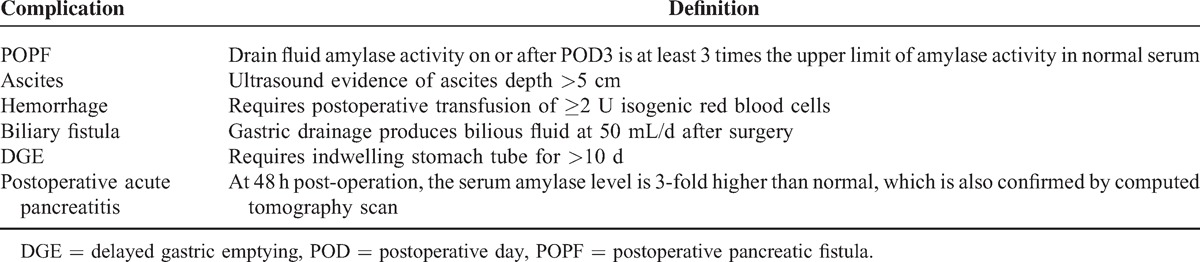
Definitions of Postoperative Complications

### Surgical Techniques and Perioperative Management

All patients underwent PD using Child procedure with an intraoperatively placed external pancreatic drainage tube. The postoperative anastomotic procedure involves end-to-end invaginating pancreaticojejunostomy with the insertion of a drainage tube into the major pancreatic duct. The drainage tube is fixed to the pancreatic parenchyma and routed out of the body via the distal bowel. We used a 5-0 nonabsorbable suture to close the posterior wall of the pancreaticojejunostomy anastomotic opening; we used a 4-0 polyethylene continuous suture to lock–stitch the anterior and posterior walls and to embed the anterior urothelium. We placed drainage tubes above and below the pancreaticojejunostomy anastomotic opening and below the biliary anastomotic opening. We treated all patients with sulbactam/cefoperazone (3.0 g/Q12 h) as preoperative prophylaxis and for the first 6 days after PD to prevent postoperative infection. We administered prophylactic intravenous octreotide at 0.6 mg/24 h for the first 3 days after surgery to decrease the rate of pancreatic secretion.

### Drain Fluid Collection and Amylase Measurement

On POD1, POD3, and POD5, we collected 5 mL gastric and pancreatic drain fluid. Gastric fluid was collected via a gastric tube that was left in the gastrointestinal anastomosis; pancreatic fluid was collected via a pancreatic drainage tube that was placed in the major pancreatic duct and supported with a stent. We measured the amylase level in the fluid within the first 30 minutes after collection using an automated biochemical analyzer (VITROS 5600; Johnson & Johnson, 100 Indiago Creek Drive Rochester, New York 14626) with VITROS Chemistry Products AMYL Slides (Johnson & Johnson). The upper limit of normal serum amylase was set at 100 U/L.

Following drain fluid collection, the sample tubes were relabeled to mask any relevant clinical information, and the amylase measurement and ratio analysis were conducted by personnel who were not aware of the patients’ identities and clinical information.

### Statistical Analysis

Statistical analyses were conducted using SPSS software (version 19.0). Data are presented as the means and 95% confidence intervals (95% CIs). We used univariate logistic regression to characterize the association between different factors and POPF. We then analyzed the factors significantly associated with POPF using multivariate analysis, which tests whether the association with a particular factor is independent of other factors. We used receiver operating characteristic (ROC) curve analysis to calculate the sensitivity, specificity, PPV, and negative predictive value (NPV), positive likelihood ratio (+LR), and negative likelihood ratio (−LR) of GPAR on POD1, POD3, and POD5. We also calculated the sensitivity, specificity, +LR, −LR, PPV, and NPV of serum and drain fluid amylase levels on POD1 using cutoffs defined previously.^5,6^ Numerical variables such as blood loss, duration of surgery, GPAR (POD1, POD3, and POD5), output volume of pancreatic drain fluid (POD1), output volume of gastric drain fluid (POD1), serum amylase level (POD1), and drain fluid amylase level (POD1) are presented as the median (Q1, Q3) in the tables.

## RESULTS

### Postoperative Complications

The study participants were recruited from August 1, 2012 to September 15, 2014. Of the 61 patients who underwent PD, 23 (38%) experienced ≥1 postoperative complications: there were 21 (34%) cases of POPF, 4 ascites, 7 hemorrhages, 1 biliary leakage, 1 DGE, and 1 postoperative acute pancreatitis (Table 2). Among the 21 patients with POPF, we classified 4 (19%), 13 (62%), and 4 (19%) as grades A, B, and C, respectively (Table 2), according to the ISGPF definition.^1^

**TABLE 2:**
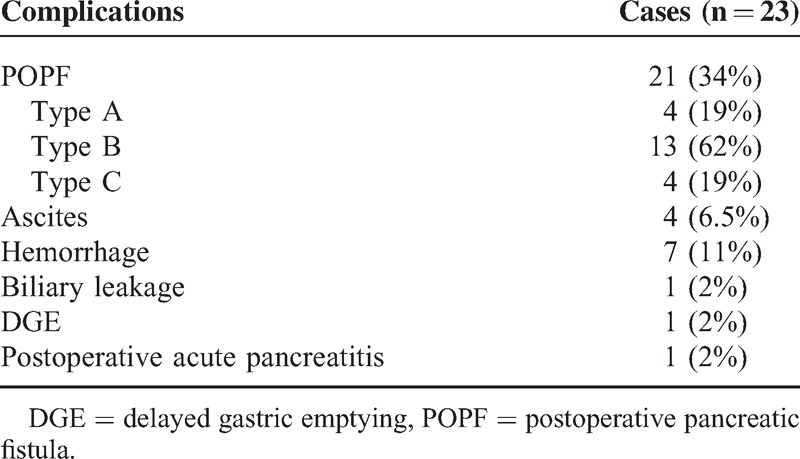
Summary of Postoperative Complications

### Association Between POPF and Clinical and Biochemical Factors

To analyze the association between POPF and various clinical and biochemical factors, we stratified the patients into groups according to POPF status (POPF and non-POPF) and compared the difference of various biomarkers between the 2 groups. All biomarkers were measured successfully, and no data from any patients were omitted in subsequent analyses. Consistent with previous reports, pancreatic consistency, pancreatic drain fluid clarity, and serum and pancreatic drain fluid amylase levels on POD1 were all significantly different between the 2 groups (Table 3). The median output of pancreatic drain fluid in the POPF group was lower than that in the non-POPF group (5 vs 20 mL, *P* = 0.01, <0.001, Table 3). Intriguingly, the median GPAR in the POPF group was significantly higher than that in the non-POPF group (POD1, 1.07 vs 0.96, X = −2.975, *P* = 0.003; POD3, 2.87 vs 0.24, X = −5.799, *P* < 0.001; POD5, 3.02 vs 0.26, X = −5.723, *P* < 0.001, Table 3). Overall, our analysis revealed that GPAR, along with the 6 other biomarkers, was significantly associated with POPF. We compared the pancreatic and gastric amylase levels between the POPF and non-POPF groups using scatter plot analysis and found no difference between them, and the amylase ranges overlapped between the 2 groups on POD1 (median pancreatic amylase in POPF group = 1435 U/L, non-POPF group = 1525 U/L; and median gastric amylase in POPF group = 1536 U/L, non-POPF group = 960 U/L).

**TABLE 3:**
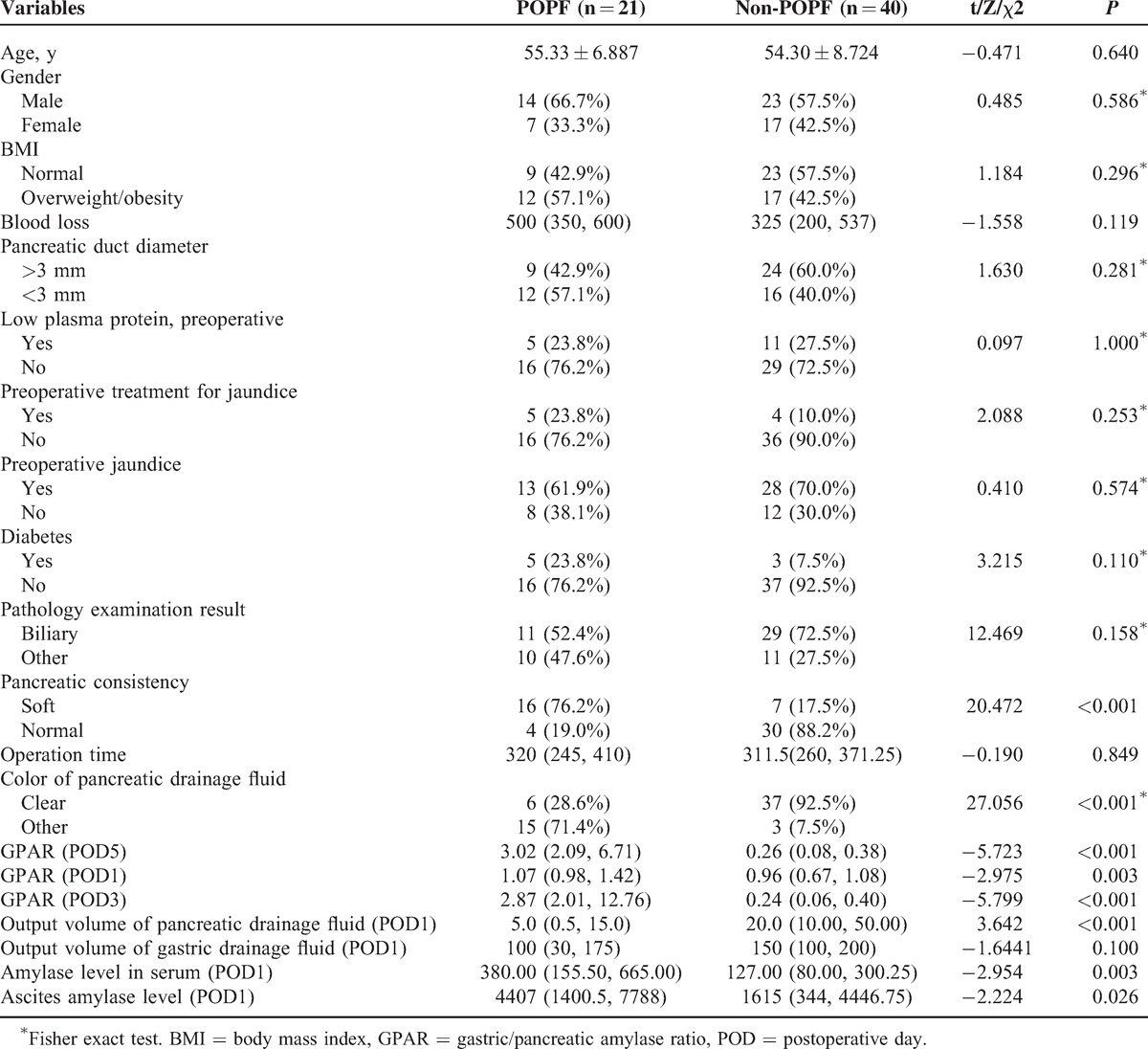
General Risk Factors for Pancreatic Leakage

### Identification of POPF Risk Factors

Next, we sought to identify the risk factors for POPF. Univariate logistic analysis determined that pancreatic consistency, pancreatic drain fluid clarity, and serum amylase levels on POD1, and GPAR on POD1, POD3, and POD5 were risk factors for POPF (Table 4). Further analysis with multivariate logistic regression confirmed that both GPAR >0.3 and turbid pancreatic drain fluid increased the risk of developing POPF (GPAR on POD3: odds ratio [OR], 70.373; 95% CI, 5.301–934.295, *P* < 0.041; turbid pancreatic drain fluid: OR, 43.341; 95% CI, 2.917–643.903, *P* = 0.006; Table 4).

**TABLE 4:**
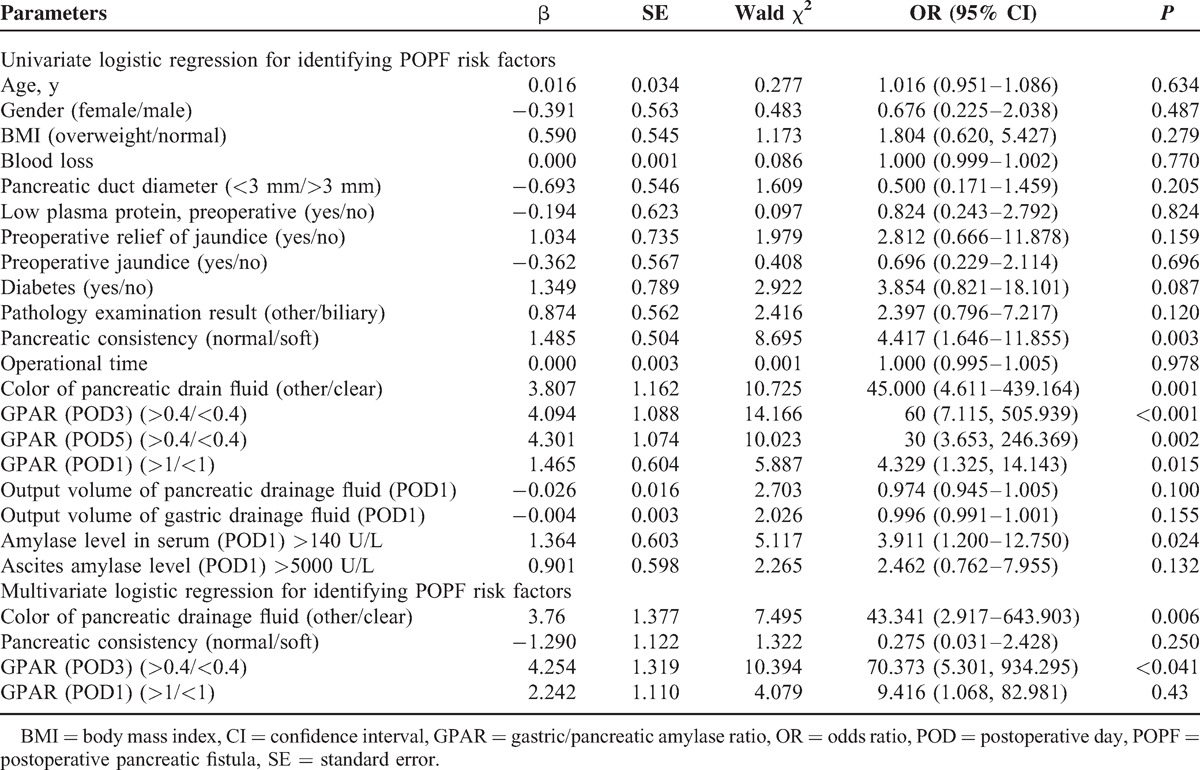
Univariate and Multivariate Logistic Regression for Identifying POPF Risk Factors

### POD3 GPAR as a POPF Predictor

**FIGURE 1:**
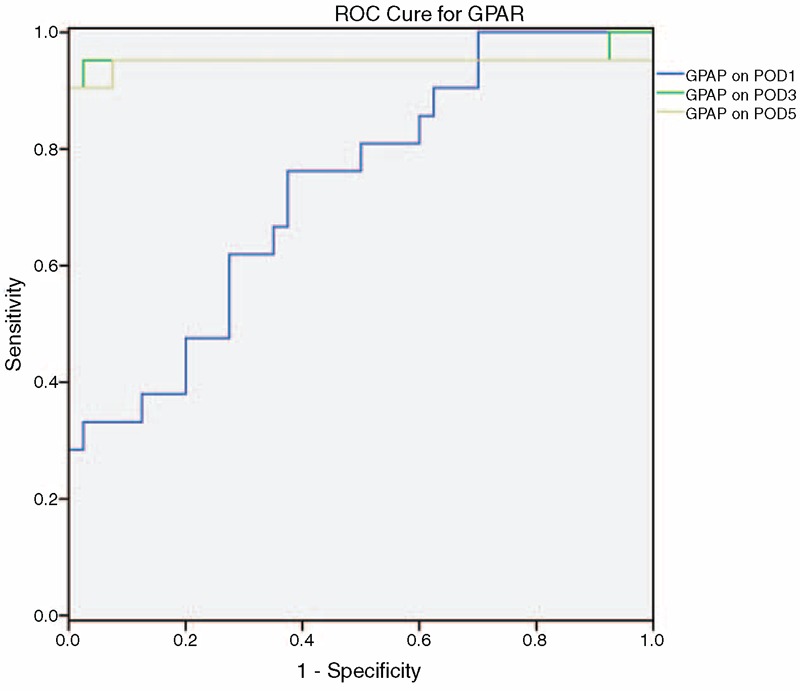
ROC curve of GPAR on POD3 for predicting POPF. ROC was performed with GPAR calculated by normalizing gastric amylase level with pancreatic amylase level on POD1, POD3, and POD5. The AUC on POD1 is 0.733 (95% CI, 0.604–0.863) with *P* = 0.003. The AUC on POD3 is 0.955 (95% CI, 0.870–1) with *P* < 0.001. The AUC POD5 is 0.949 (95% CI, 0.858–1) with *P* < 0.001. AUC = area under the curve, CI = confidence interval, GPAR = gastric/pancreatic amylase ratio, POD = postoperative day, ROC = receiver operating characteristic.

That GPAR was an independent risk factor of POPF encouraged us to further evaluate its predictive power for POPF. To this end, we conducted ROC analysis and found that with a cutoff value of 1.24, the GPAR on the POD3 curve had an area under the curve (AUC) of 0.955 (95% CI, 0.870–1, *P* < 0.001) with 90.5% sensitivity, 100% specificity, 100% PPV, and 95.2% NPV; the −LR was 0.095. We did not calculate the +LR because there were no false positives (Figure 1). We also conducted ROC analysis for GPAR at the other time points. With a cutoff value of 1, the GPAR on the POD1 curve had an AUC of 0.733 (95% CI, 0.604–0.863, *P* = 0.003) with 76.2% sensitivity, 60% specificity, 48.5% PPV, and 82.1% NPV; the +LR was 1.905 and the –LR was 0.4. With a cutoff value of 1.15, the GPAR on the POD5 curve had an AUC of 0.949 (95% CI, 0.858–1, *P* < 0.001) with 90.5% sensitivity, 100% specificity, 100% PPV, and 95.2% NPV; the −LR was 0.095 and the +LR was not calculated because there were no false positives. To compare GPAR on POD3 to previously identified markers, we evaluated the predictive power of 2 previously identified POPF predictors, that is, POD1 drain fluid and serum amylase levels, using previously defined cutoff values (drain fluid amylase: 5000 U/L; serum amylase: 140 U/L).^5,6^ POD1 drain fluid amylase level predicted POPF with 50% sensitivity, 71.1% specificity, 38.1% PPV, 80% NPV, 1.71 +LR, and 0.70 −LR; serum amylase had 76.2% sensitivity, 55% specificity, 47.1% PPV, 81.5% NPV, 1.69 +LR, and 0.43 −LR. These results suggest that GPAR on POD3 is a much more accurate POPF predictor than these 2 amylase-based markers.

## DISCUSSION

Although it was introduced by Whipple, PD has been used as a standard procedure for treating malignant and benign disorders of the pancreatic head and the periampullary region.^2^ It is considered one of the most challenging surgeries because it involves complicated procedures, requires an extended duration of surgery, and causes potentially significant tissue damage.^2^ Although anastomosis techniques have advanced greatly, the incidence of PD-associated POPF remains high.^13–15^ POPF is a major cause of morbidity and mortality in patients who undergo PD.^1–3^ Therefore, early and accurate prediction of POPF is essential for achieving optimal postoperative outcome in these patients.

We measured gastric and pancreatic drain fluid amylase levels on POD3 and calculated the GPAR in 61 patients who underwent PD. We then analyzed the association between POPF and GPAR and other biomarkers, finding that high GPAR and turbid pancreatic drain fluid are risk factors for POPF. In ROC curve analysis, the AUC of GPAR was 0.955 (95% CI, 0.87–1, *P* < 0.001) with 90.5% sensitivity, 100% specificity, 100% PPV, 95.1% NPV, and 0.095 −LR. The performance of GPAR on POD3 or POD5 was greatly superior to that of drain fluid or serum amylase levels. This is the first study to demonstrate that POD3 GPAR is a risk factor and an accurate predictor of POPF in patients who have undergone PD.

Biomarkers such as soft pancreatic consistency,^14^ BMI,^16^ pancreatic duct diameter <3 mm,^16^ serum amylase level,^17^ drain fluid amylase level,^6^ combined serum albumin and leukocyte count,^7^ combined BUN and serum albumin levels,^8^ and persisting ratio of drain fluid amylase output^9^ have been identified as POPF predictors. However, their sensitivity or accuracy tends to be low. For example, Sutcliffe et al^6^ found that low drain fluid amylase (cutoff of 350 U/L) predicted POPF with 100% and 79% sensitivity and specificity, respectively. However, the PPV was only 41%, suggesting that this method failed to identify a large portion of patients who have POPF. Similarly, Cloyd et al^5^ found that POD1 serum amylase could predict POPF, yet the PPV was only 29.3%. Here, we evaluated the performance of serum and drain fluid amylase on POD1 in predicting POPF. Consistent with previous reports, their accuracy was low: POD1 drain fluid amylase predicted POPF with 50% sensitivity, 71.1% specificity, 38.1% PPV, 80% NPV, 1.71 +LR, and 0.70 −LR; serum amylase had 76.2% sensitivity, 55% specificity, 47.1% PPV, 81.50% NPV, 1.69 +LR, and 0.43 −LR. In comparison, POD3 GPAR predicted POPF with greater accuracy, with 92.9% sensitivity, 100% specificity, 100% PPV, and 96.92% NPV. In fact, POD3 GPAR predicted POPF in 19 of the 21 patients with POPF. One patient whose diagnosis was missed developed POPF 14 days after PD; his BUN and serum albumin levels at POD10 were 16.41 mmol/L and 29.8 g/L, respectively. Therefore, POPF in this patient may not have resulted from anastomosis failure, but rather from malnutrition and systemic nitrogen imbalance.

**FIGURE 2:**
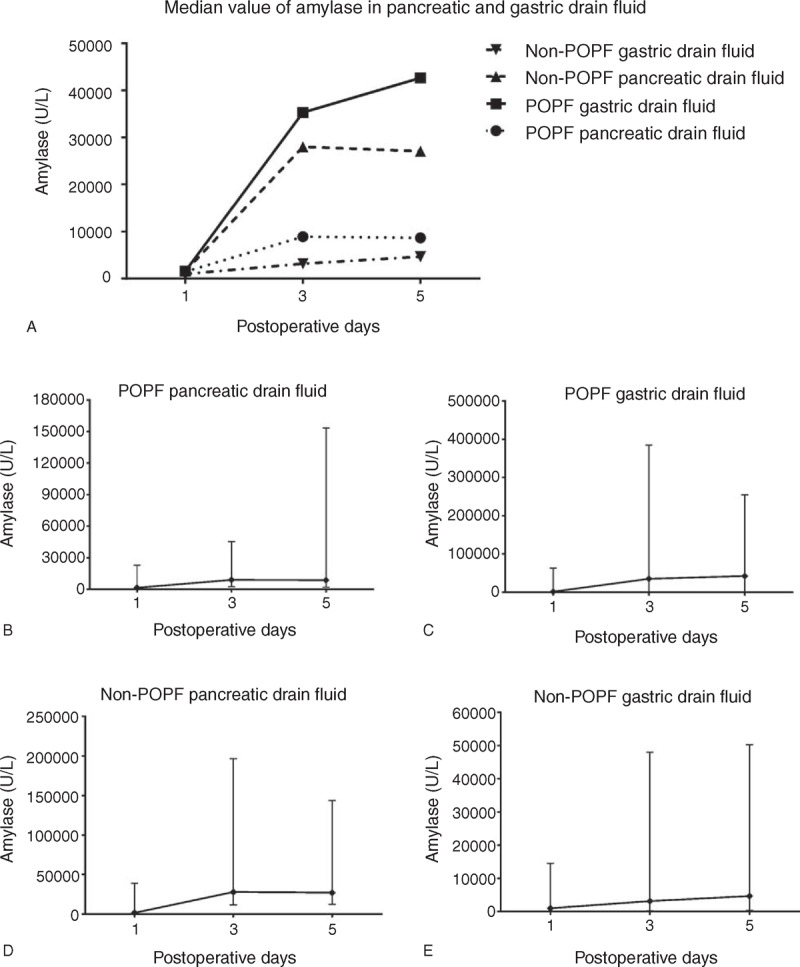
Schematic diagram of 2 different scenarios for pancreatic fluid flow. (A) Pancreatic fluid flows into the gastric tube and (B) pancreatic fluid flows out through the pancreatic drain tube. When pancreatic fluid is not drained through pancreatic drainage tube or is predominately from the pancreatic stump, it will get into gastrointestinal anastomosis and lower gastrointestinal tract, so the amylase in gastric fluid would increase and the amylase in pancreatic drainage tube would decrease (A). On the other hand, when pancreatic fluid is drained through pancreatic drainage, the amylase level in the pancreatic drain fluid increases, while that in the gastric drain fluid decreases (B).

We reasoned that a major challenge in predicting POPF using amylase levels is that pancreatic exocrine function can vary dramatically between patients, and such variation influences the baseline and postoperative amylase levels significantly. In the present study, while the POD3 pancreatic amylase level in the non-POPF group ranged 11,433–554,050 U/L, it ranged 2307–45,318 U/L in the POPF group, demonstrating that amylase levels in pancreatic drainage vary dramatically between patients and that the absolute amylase level did not correlate with POPF. To explain this observation, we propose that GPAR may serve as an indicator for the direction of pancreatic fluid flow: when pancreatic fluid is not drained through the pancreatic drainage tube or is predominately from the pancreatic stump, it will infiltrate the gastrointestinal anastomosis and lower gastrointestinal tract, resulting in high GPAR and causing POPF; when pancreatic fluid is drained from the body through the external drainage tube, it does not flow through the gastrointestinal anastomosis and lower gastrointestinal tract, therefore the GPAR value is low and the surrounding tissues are unharmed (Figure 2). A similar mechanism can also be applied to explain the correlation in the color change in the pancreatic fluid.

**FIGURE 3:**
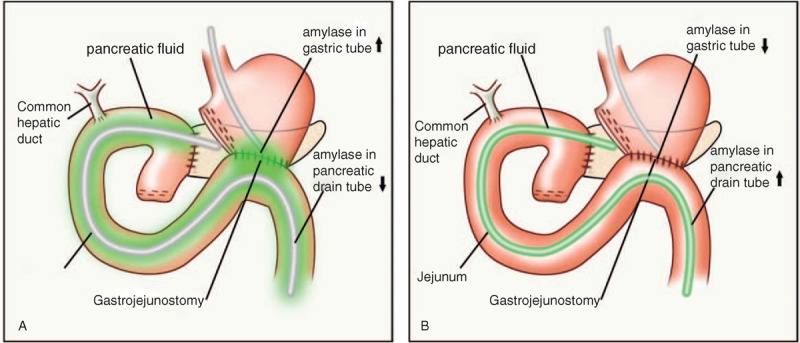
(A) Median value of amylase in pancreatic and gastric drain fluid. Scatter plots of amylase levels in pancreatic and gastric drain fluid in (B and C) POPF and (D and E) non-POPF groups. Although the pancreatic and gastric drain fluid amylase levels were not at their apex, their values reflected the trend of change. POPF = postoperative pancreatic fistula.

The reasons for our use of POD3 as the time point for POPF prediction are as follows. As shown in the scatter plot in Figure 3, the pancreatic and gastric fluid amylase levels on POD1 were low and there was no difference between the POPF and non-POPF groups. By contrast, the amylase levels on POD3 were at their apex and therefore correlated well with POPF. As there was a significant amount of postoperative tissue response, and because postoperative medication significantly influences amylase levels immediately after surgery, POD1 may not be the best time point to make accurate predictions of POPF. Conversely, as postoperative tissue response and medication decrease over time, POD3 is a better time point for measuring the levels of biochemical parameters to predict POPF. For example, the patient with the highest pancreatic amylase activity (55,4050 U/L) had gastric drain fluid amylase activity of 23,1242 U/L, therefore a GPAR of 0.42, and developed no POPF. By contrast, a patient with low pancreatic amylase activity (7123 U/L) had relatively high gastric drain fluid amylase activity (38,4621 U/L); the GPAR was 54, and the patient developed POPF. Although the GPAR on POD5 also predicted POPF well, predicting POPF on POD5 is too late because most POPF occurs between POD5 and POD7.^18^ Altogether, our results suggest that POD3 is the best time point for predicting POPF using the GPAR.

It is worth noting that as the patients who undergo PD often have the pyloric sphincter removed and subsequently salivary amylase may be present in gastric drainage. As both salivary and pancreatic amylase belong to the α-amylase family, the detection method currently used in most clinical settings cannot distinguish the two. Thus, both pancreatic and salivary amylases contribute to the amylase activity in gastric drain fluid. However, as the maximal normal salivary amylase activity is 454.125 ± 332.008 U/L in men and 457.142 ± 367.517 U/L in women,^19^ we believe that the influence of salivary amylase is not a significant concern in the present study.

In addition to GPAR, we also identified turbid pancreatic drain fluid as a risk factor for POPF. Soft pancreatic consistency and pancreatic tube diameter have been reported as POPF risk factors,^17,20,21^ yet our multivariate analysis identified only turbid pancreatic drain fluid as a risk factor for POPF. As pancreatic consistency and pancreatic tube diameter may be influenced by POPF, they may not be reliable predictors of POPF; however, the predictive power of these clinical factors has not been explored. To our knowledge, our study is the first to describe turbid pancreatic drain fluid as a risk factor for POPF. Our findings warrant further investigations to determine whether turbid pancreatic drain fluid can predict POPF.

In summary, we conducted a prospective study of 61 patients who underwent PD and identified POD3 GPAR as a factor significantly associated with POPF; with a cutoff of 1.24, it predicts POPF with high sensitivity and specificity. Measuring pancreatic and gastric drain fluid amylase activity is straightforward and inexpensive. We believe that the promising results reported warrant further evaluation in a much larger patient population to confirm the predictive power of POD3 GPAR for POPF and determine the optimal cutoff value.

## References

[1] Bassi C Dervenis C Butturini G Postoperative pancreatic fistula: an international study group (ISGPF) definition. *Surgery* 2005; 138:8–13. 16003309 10.1016/j.surg.2005.05.001

[2] Ho CK Kleeff J Friess H Complications of pancreatic surgery. *HPB (Oxford)* 2005; 7:99–108. 18333171 10.1080/13651820510028936 PMC2023932

[3] Oneil Machado N Pancreatic fistula after pancreatectomy: definitions, risk factors, preventive measures, and management-review. *Int J Surg Oncol* 2012; 2012:602478. 22611494 10.1155/2012/602478 PMC3348641

[4] Pratt WB Maithel SK Vanounou T Clinical and economic validation of the International Study Group of Pancreatic Fistula (ISGPF) classification scheme. *Ann Surg* 2007; 245:443–451. 17435552 10.1097/01.sla.0000251708.70219.d2 PMC1877022

[5] Cloyd JM Kastenberg ZJ Visser BC Postoperative serum amylase predicts pancreatic fistula formation following pancreaticoduodenectomy. *J Gastrointest Surg* 2014; 18:348–353. 23903930 10.1007/s11605-013-2293-3

[6] Sutcliffe RP Battula N Haque A Utility of drain fluid amylase measurement on the first postoperative day after pancreaticoduodenectomy. *World J Surg* 2012; 36:879–883. 22354484 10.1007/s00268-012-1460-0

[7] Kawai M Tani M Hirono S How do we predict the clinically relevant pancreatic fistula after pancreaticoduodenectomy? An analysis in 244 consecutive patients. *World J Surg* 2009; 33:2670–2678. 19774410 10.1007/s00268-009-0220-2

[8] Relles DM Richards NG Bloom JP Serum blood urea nitrogen and serum albumin on the first postoperative day predict pancreatic fistula and major complications after pancreaticoduodenectomy. *J Gastrointest Surg* 2013; 17:326–331. 23225108 10.1007/s11605-012-2093-1

[9] Okano K Kakinoki K Suto H Persisting ratio of total amylase output in drain fluid can predict postoperative clinical pancreatic fistula. *J Hepatobiliary Pancreat Sci* 2011; 18:815–820. 21594559 10.1007/s00534-011-0393-6

[10] Sikkens EC Cahen DL de Wit J Prospective assessment of the influence of pancreatic cancer resection on exocrine pancreatic function. *Br J Surg* 2014; 101:109–113. 24338808 10.1002/bjs.9342

[11] Williams ST Woltering EA O’Dorisio TM Effect of octreotide acetate on pancreatic exocrine function. *Am J Surg* 1989; 157:459–462. 2469337 10.1016/0002-9610(89)90634-x

[12] Bassi C Butturini G Molinari E Pancreatic fistula rate after pancreatic resection. The importance of definitions. *Dig Surg* 2004; 21:54–59. 14707394 10.1159/000075943

[13] Bassi C Falconi M Molinari E Duct-to-mucosa versus end-to-side pancreaticojejunostomy reconstruction after pancreaticoduodenectomy: results of a prospective randomized trial. *Surgery* 2003; 134:766–771. 14639354 10.1016/s0039-6060(03)00345-3

[14] Kingsnorth AN Duct to mucosa isolated Roux loop pancreaticojejunostomy as an improved anastomosis after resection of the pancreas. *Surg Gynecol Obstet* 1989; 169:451–453. 2814757

[15] Xiong JJ Altaf K Mukherjee R Systematic review and meta-analysis of outcomes after intraoperative pancreatic duct stent placement during pancreaticoduodenectomy. *Br J Surg* 2012; 99:1050–1061. 22622664 10.1002/bjs.8788

[16] El Nakeeb A Salah T Sultan A Pancreatic anastomotic leakage after pancreaticoduodenectomy. Risk factors, clinical predictors, and management (single center experience). *World J Surg* 2013; 37:1405–1418. 23494109 10.1007/s00268-013-1998-5

[17] Frymerman AS Schuld J Ziehen P Impact of postoperative pancreatic fistula on surgical outcome – the need for a classification-driven risk management. *J Gastrointest Surg* 2010; 14:711–718. 20094814 10.1007/s11605-009-1147-5

[18] Blumgart LH Blumgart's Surgery of the Liver, Biliary Tract and Pancreas. 4th ed 2012; 893–897.

[19] van den Bos R Taris R Scheppink B Salivary cortisol and alpha-amylase levels during an assessment procedure correlate differently with risk-taking measures in male and female police recruits. *Front Behav Neurosci* 2013; 7:219. 24474909 10.3389/fnbeh.2013.00219 PMC3893681

[20] Hashimoto N Ohyanagi H Effect of acute portal hypertension on gut mucosa. *Hepatogastroenterology* 2002; 49:1567–1570. 12397737

[21] Lee SE Yang SH Jang JY Pancreatic fistula after pancreaticoduodenectomy: a comparison between the two pancreaticojejunostomy methods for approximating the pancreatic parenchyma to the jejunal seromuscular layer: interrupted vs continuous stitches. *World J Gastroenterol* 2007; 13:5351–5356. 17879405 10.3748/wjg.v13.i40.5351 PMC4171325

